# Principles of Microsurgery for Experimental Urethroplasty

**DOI:** 10.3390/jcm15145362

**Published:** 2026-07-09

**Authors:** Fabio Campodonico, Emanuela Ognio, Antonella Brizzolara, Anna Favre, Vittoria Campodonico, Giorgio Carmignani

**Affiliations:** 1 Urology Unit, Ospedale Galliera, 16128 Genova, Italy; 2 National Institute for Cancer Research, 16132 Genova, Italy; emanuela.ognio@alice.it; 3 IRCCS San Martino Hospital, 16132 Genova, Italy; antonella.brizzolara@iit.it (A.B.); giocarmi@gmail.com (G.C.); 4 Giannina Gaslini Children’s Hospital, University of Genova, 16147 Genova, Italy; annafavre@libero.it; 5 Sapienza Medical School, University of Rome, 00161 Rome, Italy; vittoriacampodonico1@gmail.com

**Keywords:** urethroplasty, animal models, rat, rabbit, microsurgery, reconstructive urology

## Abstract

Preclinical studies using animal models are of utmost importance for translating innovative interventions from experimental to clinical settings. However, relatively few experimental urethroplasty studies have been conducted, and the number of randomized controlled trials remains limited; consequently, the overall quality of reporting in this field is still suboptimal. There is therefore a need to establish reproducible experimental models based on a sound understanding of laboratory animal anatomy and physiology, combined with refined surgical techniques appropriate for each species. For the rabbit model, a more equipped environment is needed, set up in such a way as to allow surgery to be performed in a sterile field or in any case with a very low bacterial load. A replicable model for experimental urethroplasty was proposed, including ventral onlay urethroplasty using a free autologous graft. The fascia lata was selected as the graft material owing to its ease of harvest, the quantity available, and the absence of significant donor-site morbidity. The rat model, instead, requires a microscope and microsurgical instruments. Although the rat is not traditionally considered a standard experimental model for urethral surgery—mainly due to the technical difficulty imposed by its small size—it can serve as a reliable model when proper microsurgical training is applied. Three key technical points are crucial for successful surgery: control of urethral bleeding by proximal compressive hemostasis; meticulous handling of the graft, ensuring accurate suturing once it is fully spread; and precise placement of sutures using refined microsurgical techniques.

## 1. Introduction

Microsurgery performed on animal models represents a valuable research and training field. The laboratory animal is not merely a model for learning basic surgical techniques, understanding tissue characteristics, and mastering the correct use of sutures; it is, above all, a living being requiring care and attention, without which high-quality research outcomes cannot be achieved. Thus, the animal serves simultaneously as the object of surgical practice and as the subject of rigorous assessment to validate experimental results. It is no coincidence that the motto *“quality of animal care = quality of science”* was coined. Within this context, it is essential to highlight the priority of ethical considerations, which must accompany every form of laboratory research. Animals must be protected under the supervision of an ethics committee, which is responsible not only for reviewing and approving research projects but also for monitoring their progress in detail at every stage, ensuring that experimental procedures are conducted correctly and ethically. Urethroplasty was first developed during the 1950s and 1960s, initially through direct anastomosis or as substitution urethroplasty for longer strictures [1]. Early substitution techniques employed skin grafts or fasciocutaneous flaps derived from penile skin [2,3,4]. The introduction of buccal mucosa as a graft material represented a major advance, simplifying the procedure and contributing to its widespread adoption among urologists [5]. From the 1990s onwards, tissue engineering has played an increasing role in the search for suitable urethral substitute materials, both natural and synthetic—including collagen, acellular matrices, small intestinal submucosa, bladder acellular matrix, and polyglycolic acid scaffolds [6]. Indeed, in order to improve the results of reconstructive surgery, the replacement with an equivalent engineered tissue avoids harvesting procedures and would improve the outcomes of patients. Biomaterials in urology function as scaffolds or templates which, making contact with the host tissue, permit proliferation and delivery of the native tissue layers and cells. Three classes of biomaterials are mainly used in reconstructive urology: collagen and alginate (naturally derived), acellular matrices, and synthetic polymers. All of them have been approved by the FDA (United States Food and Drug Administration).

Preclinical studies using animal models are of utmost importance for translating innovative interventions from experimental to clinical settings. However, relatively few experimental urethroplasty studies have been conducted, and the number of randomized controlled trials remains limited; consequently, the overall quality of reporting in this field is still suboptimal [7]. There is therefore a need to establish reproducible experimental models based on a sound understanding of laboratory animal anatomy and physiology, combined with refined surgical techniques appropriate to each species.

## 2. The Experimental Laboratory

**The team**. To carry out a research program of surgical interventions, it is essential to first create a coordinated group of collaborators able to meet all the needs that may arise, both inside the operating room and later when the animals are kept under observation for several weeks or months. The subjects involved are the following:

1. The operator: a resident or a specialist in urology, preferably trained in microsurgical techniques.
2. The veterinarian: working in the animal house where the operation is performed, induces and assists with the anesthesia, performs health checks on the animals before and after the interventions, performs euthanasia if needed.
3. The biologist: during the interventions performs blood tests and can replace the veterinarian.
4. The pathologist: in the animal house is available for the analysis of biopsies and histological examinations and produces photographic documentation.
5. Radiology facility: for radiological control after surgery when needed.

For small animal procedures (e.g., rats or mice) performed under the operating microscope, the presence of the microsurgeon—occasionally with an assistant—is generally sufficient. However, operations involving rabbits typically require a larger team: a microsurgeon, an assistant (using either a microscope or surgical loupes), and a collaborator responsible for anesthesia and the sterile field.

**The operating room and the instruments**. The organization of an operating room can be simple when small animals such as rats or mice must be operated on. For these animals, sterility is not essential [8]. Their remarkable resistance to infections allows the advantage of being able to quickly set up an operating table and above all to avoid septic complications even when performing dirty surgery such as intestinal surgery without antibiotic prophylaxis. Therefore, it is not necessary to have a laboratory similar to a typical operating room, but a room equipped with an operating microscope and adequate instruments is sufficient.

In our practice, we have set up a room dedicated exclusively to small animal surgery. It is equipped with an Olympus microscope mounted on a floor stand, with electric motor zoom and focus with pedal control, double binoculars and connection for camera and video. In the same room, the animal is weighed, induced to anesthesia and shaved for surgery. During the awakening interval, it is kept under a heat lamp and then placed in the cage.

**Figure 1:**
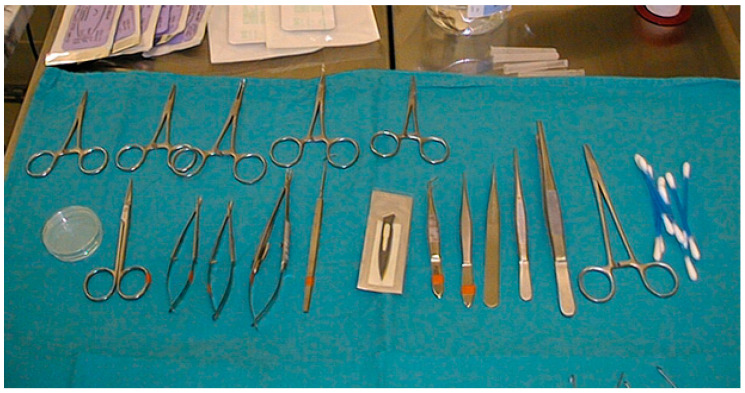
Surgical instruments for microsurgery.

Microsurgical instruments are essential, but a traditional delicate surgery set is necessary to be able to quickly complete the preliminary phases without a microscope (Figure 1). The sutures must be prepared according to the needs and type of intervention; thus, a supply of 6-0, 8-0, 9-0, and 10-0 sutures is useful.

Essential instruments for microsurgery [9,10,11]:

- Jeweler’s forceps no. 3 and 5.
- Curved-tip tweezers.
- Thin-tip needle holder (Castroviejo model).
- Straight and curved-tip microsurgical scissors (Vannas model).
- Small surgical scissors.
- An Acland-type microsurgical approximator.
- Two microsurgical vascular clamps.
- Small retractors (also handmade).

**Figure 2:**
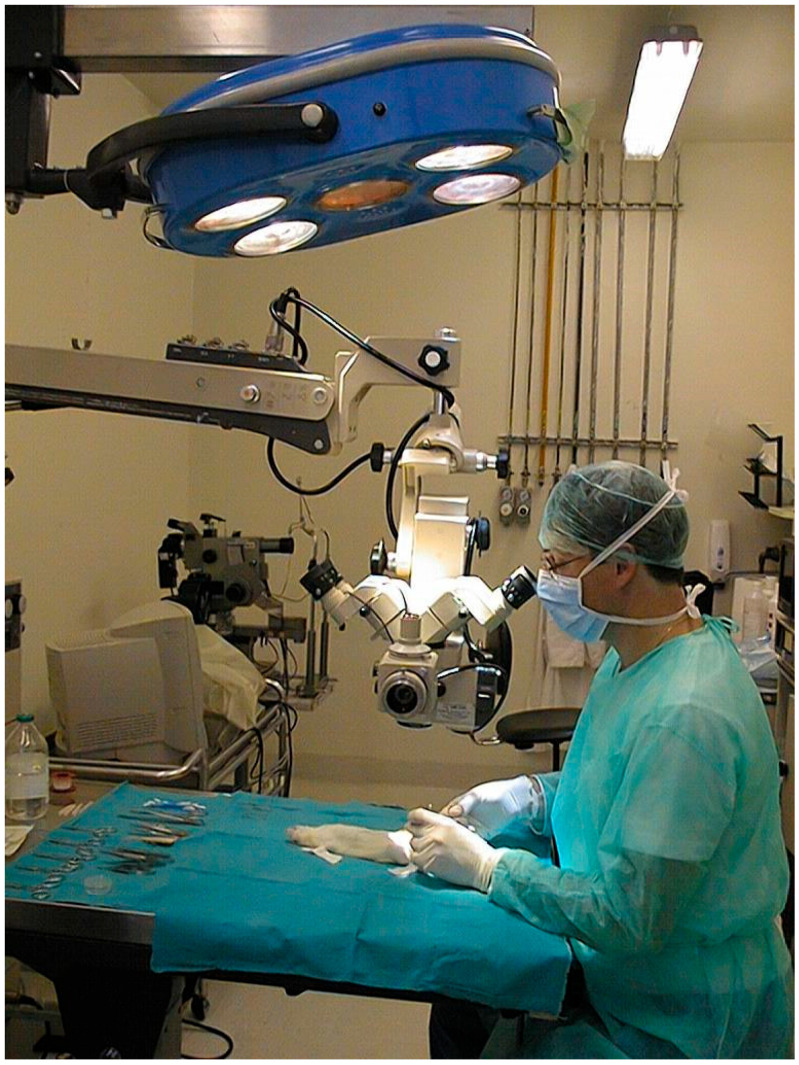
Operating room for experimental microsurgery. The surgical table is suitable for sitting or standing surgery.

For the rabbit, two considerations must be made: first, a more equipped environment is needed, set up in such a way as to allow surgery to be performed in a sterile field or in any case with a very low bacterial load. Secondly, for some operations, not only in laparotomies, it is possible to operate standing up and with more than one person because the size of the animal is comparable to that of a newborn. This often requires an operating room with a suitable operating table. In our experience, we used a room equipped and dedicated to the rabbit, including an operating microscope, a high table with a surgical lamp, a sterilizer for surgical instruments, drapes and gauze, and a monopolar electrocautery (Figure 2).

### 2.1. Anesthesia and Analgesia

In the rat, pre-anesthesia is performed according to the following scheme:

- Atropine 1.5 mg/kg i.p. (intraperitoneal).
- After 30 min: Diazepam 3–6 mg/kg i.p.
- After about 10 min, Xylazine 5–10 mg/kg i.m. (intramuscular).

Anesthesia is performed with ketamine 35–44 mg/kg i.m. after about 10–15 min.

This schedule has allowed us to perform any type of intervention up to a duration of about 3 h (with the addition of ketamine boluses).

In the mouse, the sequence is the same but slight differences concern the doses.

In the rabbit we performed only accesses to the pelvis and interventions on the urethra for which chemical anesthesia was sufficient, according to the following schedule:

- Diazepam 1.7 mg/kg i.m.
- After about 10 min, Xylazine 4–6 mg/kg i.m.
- After about 10 min, anesthesia with Ketamine 35–50 mg/kg i.m.

Regarding analgesia in the postoperative phase, mice and rats are treated with acetylsalicylic acid dissolved in water at doses of 20 mg/kg and 100 mg/kg, respectively.

In rabbits, we use 1 vial of 10 mg ketorolac possibly followed by a second or third dose at 6 h intervals.

### 2.2. Surgical Anatomy of the Laboratory Animal

#### 2.2.1. The Genitourinary Tract in Male Rat

**The bladder**: protrudes into the abdominal cavity above the upper margin of the pubis. It is pear-shaped, about 10 mm long and 5 mm wide at the level of the dome. When full, it acquires a spherical configuration of about 25 × 15 mm or more and is transparent. It has a mucosa with plications, multilayered transitional epithelium and a thin muscular coat. It is covered by peritoneum. The ventral prostatic lobe surrounds the bladder anteriorly and laterally.

**Figure 3:**
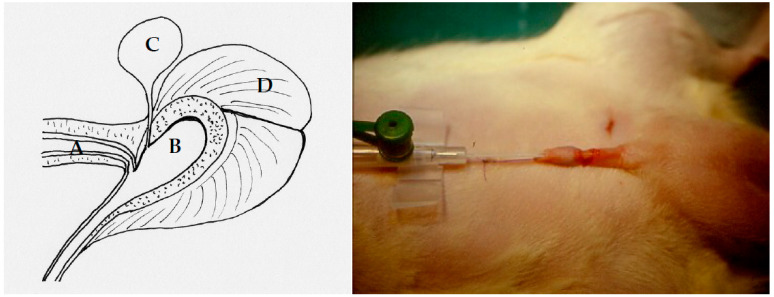
Critical point of the bulbar urethra. A, pelvic urethra; B, urethral diverticulum; C, bulbourethral gland; D, bulbocavernosus muscle. In the photo, the cannula has been positioned up to the urethral diverticulum after fixing the penis in tension with a stitch on the abdomen. The incision for degloving is subcoronal.

**The urethra**: has a variable caliber in its different segments. In the pelvic tract it measures 0.7 mm in diam., which progressively reduces because along the dorsal wall the seminal colliculus protrudes significantly into the lumen until it joins at the bulbar level with two lateral folds that form two diverticula lateral to the colliculus [12]. For this reason, it is not possible to perform an atraumatic retrograde catheterization beyond the bulb (Figure 3). The bulb is surmounted by the bulbocavernosus muscle and houses the orifices of the bulbourethral glands. Other minor acinar glands with short independent ducts, called urethral glands, are located in the cavernous layer of the pelvic and bulbar tract. From this point, the penile urethra continues without obstacles to the glans, hidden by the preputial pouch in the flaccid state. The bulbo-penile urethra is lined by an epithelium that overlies the corpus spongiosum. The muscles that surround the urethra are the bulbocavernosus, ischiocavernosus and the external anal sphincter located behind the bulbocavernosus.

**Operation**: it can be cannulated with small soft 22- and 20-gauge probes but only up to the bulb for the above-mentioned reason. The isolation of the urethra is easy after incision of the skin and penile fascia. Exposure of the urethra, however, is difficult due to the lack of a constant avascular line on the corpus spongiosum and bleeding is almost constant, but this obstacle can be overcome by placing a hemostatic strip at the base of the penis.

**The penis**: in the pendulous portion it is about 2.5 cm long. It has a pronounced ventral concavity at the mid-level (flexura penis). The suspensory ligament consisting of a strip of deep fascia connects it to the abdominal wall. The corpus cavernosum is contained within a resistant double layer of tunica albuginea. Distally, the tunica albuginea layer joins the penile bone, a small osteocartilaginous body contained in the glans. Above the corpus cavernosum there is the penile fascia.

#### 2.2.2. The Genitourinary Tract in Male Rabbit

**The bladder**: similarly to that of the rat, it is pear-shaped but much larger in size. When opened, it is possible to distinguish fairly well macroscopically the mucosa from the muscular coat, the ureteral meatuses and the internal urethral meatus that define a trigone. It is possible to cannulate the ureters retrogradely with a normal 2 French ureteral catheter on a guide wire.

**The urethra**: it is divided into prostatic, membranous and spongy portions. The prostatic urethra, as in humans, is located inside the gland of the same name but receives the outlet of both the prostatic ducts and some paraprostatic glands. The membranous urethra is short and receives the outlet of the two bulbourethral glands. The spongy urethra extends from the bulb to the tip of the penis [13,14]. Histologically, the urethra is lined by a transitional epithelium.

**Operation**: it can be easily catheterized retrogradely with an 8 Fr Foley, but sometimes, especially in smaller animals, a guide is necessary. In our experience, an 8 Fr Baxter K30 nasogastric tube with a rounded tip has been very useful because it has a larger internal diameter, which is essential because the catheter tends to block easily due to the high density of the rabbit’s urine.

Exposure of the urethra is possible by performing an easy degloving, incising the penile fascia and sectioning the corpus spongiosum along the thin median avascular line.

**The penis**: the pendulous portion is 3–4 cm long and includes the spongy urethra outside the pelvic canal. In the distal part, the glans is contained in a preputial pocket. Below the glans there is a small bone called “os penis” or baculum.

## 3. Experimental Urethroplasty Model in the Rabbit

**Figure 4:**
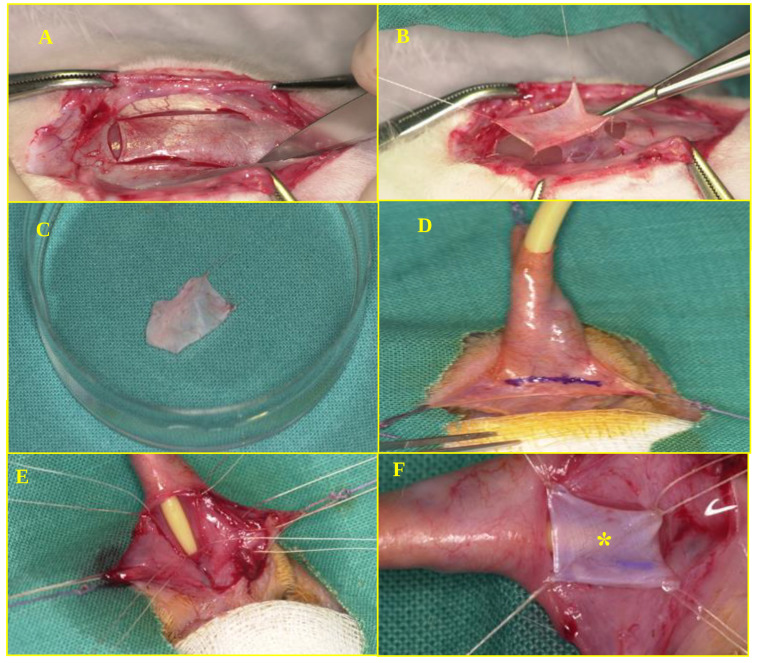
(**A**) Harvesting of the fascial graft. (**B**) The graft is carefully brought up along its epimysium layer. (**C**) The graft is put in a Petri dish with saline. (**D**) The rabbit urethra is marked for incision. (**E**) The ventral urethra is open and exposed. (**F**) The graft is laid on the ventral urethral defect with correct polarity using a blue line mark (*).

**Figure 5:**
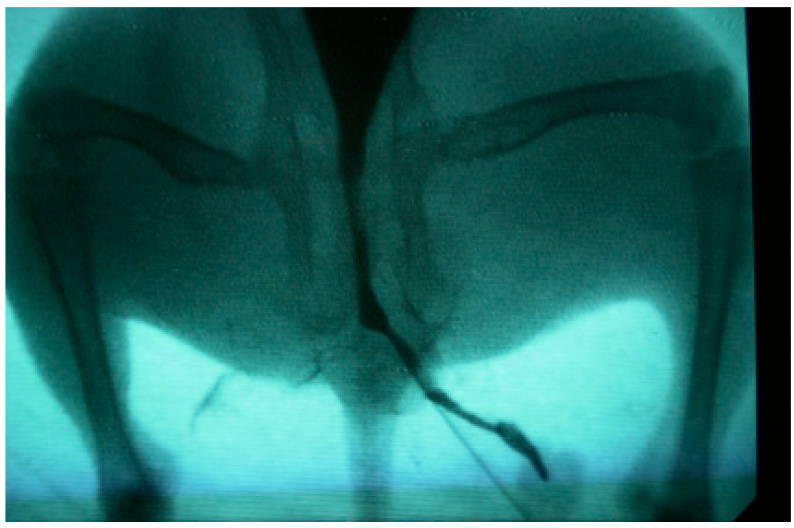
Postoperative radiological control. After filling the bladder with contrast medium solution, a guide wire was inserted to stimulate the urinary reflex. The antegrade urethrography shows a regular urethral shape.

A validated model for experimental urethroplasty is the ventral onlay urethroplasty using a free autologous graft. The fascia lata is selected as the graft material owing to its ease of harvest, the quantity available, and the absence of significant donor-site morbidity [15]. The fascia lata constitutes the deep fascial layer of the connective tissue system covering the thigh musculature. It consists of distinct bundles of collagen fibers predominantly aligned parallel to the muscular axis, interspersed with elastic fibers and mature fibroblasts [16]. Its vascular supply is derived mainly from the superficial fascio-cutaneous system, with numerous veins draining into the saphenous circulation [17]. The fascia lata demonstrates excellent tensile strength, elasticity, and smoothness, providing a relatively frictionless surface over the underlying epimysium. The correct anatomical and surgical dissection plane lies within the thin, avascular layer of areolar tissue between the fascia lata and the epimysium. A male New Zealand rabbit weighing approximately 2.5 kg, after 12 h of fasting, undergoes pre-anesthesia with diazepam followed by intramuscular xylazine, and induction with intramuscular ketamine. The animal is prepared by shaving the genital region and the lateral thigh, fitting an Elizabethan collar, and disinfecting the shaved skin with povidone–iodine. Through a transverse incision of the superficial thigh layers, the deep fascia (fascia lata) is identified and a rectangular patch of approximately 1 × 2 cm is excised, elevated beneath the areolar interface between fascia and muscle, taking care to avoid trauma to the delicate laminar structures. The graft is then immersed in sterile saline solution. A Foley 8-Fr catheter is inserted into the bladder, and the degloving incision line is marked with a dermographic pen (Figure 4). Using 2.5× magnification loupes, the skin and penile fascia are incised, followed by careful sectioning of the spongy tissue along the thin midline avascular plane. The urethra is opened ventrally, and a rectangular mucosal flap is raised. The mucosal edges are exteriorized using symmetrical stay sutures of absorbable monofilament 6-0. The graft, trimmed to the appropriate size, is then positioned over the urethral defect, maintaining its natural polarity. The stay sutures are anchored at the four corners of the graft to ensure tension-free placement and enable a continuous running suture. The fascial and cutaneous layers of the penis are then closed sequentially. The catheter is maintained in situ for at least 48 h. Antibiotic prophylaxis, initiated at the time of surgery, is continued for five days (gentamicin 5 mg/kg i.m.). Animals are monitored daily, with particular attention to micturition and wound healing at the penile site. After two weeks, radiological evaluation is performed by filling the bladder with contrast medium and stimulating micturition via a guidewire to obtain a voiding urethrogram (Figure 5). To assess for fistula formation, methylene blue is injected into the urethra, and urethral calibration is carried out using a catheter to exclude stenosis. After a variable postoperative interval of several weeks, the penis is harvested for histological evaluation. Tissue sections are stained with hematoxylin and eosin and examined under light microscopy. The graft integration (*engraftment*) process occurs over approximately 72 h [18]. The initial phase, termed imbibition (within 48 h), relies on diffusion of nutrients from adjacent tissues, as vascular perfusion is not yet established and the graft remains slightly cooler. The second phase, inosculation, coincides with the onset of angiogenesis [19], resulting in revascularization of the graft.

In ten animals that underwent onlay urethroplasty, neither death nor wound infection was observed. Two fistulas were revealed at urethrography in the 1st and 3rd operated rabbits. After sacrifice, no urethral stenosis or diverticula were observed. In eight rabbits, histology showed the preservation of the original laminar bilayered structure of the patch with absence of fibrosis, and a new line of urothelium developed in all animals [18].

## 4. Experimental Urethroplasty Model in the Rat

**Figure 6:**
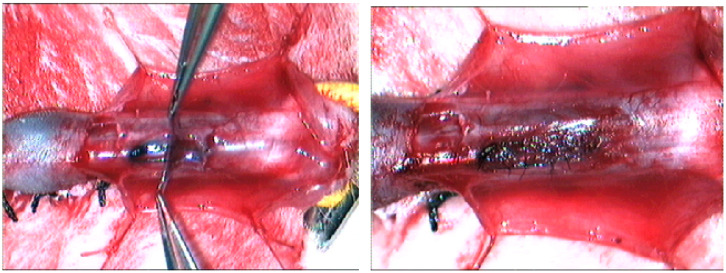
The urethral mucosa is marked with methylene blue. The peritoneal graft is laid on the urethral defect and sutured with 10/0 stitches.

A Sprague–Dawley rat, weighing 250–300 g, is anesthetized under general anesthesia. After shaving and preparing the genital and lower abdominal skin, a hypogastric laparotomy is performed and micro-retractors are positioned to expose the surgical field. The parietal peritoneum is detached by injecting physiological saline between the serosal and endo-abdominal fascial layers, creating sufficient separation to excise an adequate patch of tissue. The abdominal wall is then sutured in two layers (peritoneo-fascial and cutaneous), except at the lower margin, where a vesicostomy is constructed to protect the subsequent urethroplasty. The anterior urethra is catheterized using a 22-G intravenous cannula, and the penis is fixed under gentle traction to the abdominal wall with a single suture. The urethra is isolated for a short segment after incising the preputial skin and penile fascia, and is looped with two 3-0 stay sutures. Under the operating microscope, the corpus spongiosum is incised, and, depending on the degree of bleeding, a hemostatic strip is placed at the penile base. The peritoneal graft is then positioned over the urethral defect, trimmed to approximately 5 × 3 mm, and sutured continuously with 10-0 nylon (Figure 6). The penile fascia and skin are closed with 6-0 absorbable sutures, and the urethral cannula is removed. Integrity of the repair is verified by injecting methylene blue into the urethra to detect fistulae, followed by urethral calibration with a fine cannula to exclude stenosis. The first use of peritoneum as a urethral graft was reported by Shaul in the rabbit model [20], while Nanni [21] confirmed its feasibility in similar experimental settings. The peritoneum is a simple, easily harvested monolayer of cells, making it an attractive graft material for urethral reconstruction in the rat. Although the rat is not traditionally considered a standard experimental model for urethral surgery—mainly due to the technical difficulty imposed by its small size—it can serve as a reliable model when proper microsurgical training is applied. Three key technical points are crucial for successful surgery: control of urethral bleeding by proximal compressive hemostasis; meticulous handling of the graft, ensuring accurate suturing once it is fully spread; and precise placement of sutures using refined microsurgical techniques. Moreover, the presence of a vesicostomy is essential, as maintaining a urethral catheter in an awake rat is challenging, unlike in the rabbit [22].

## 5. Ethical Principles

When research is based on animal testing, ethical problems move together with scientific ones; in other words, they are strictly interdependent. Ethics is based on morality. The moral agent is the man, that is, the subject capable of making moral choices. The moral object is the animal, a being worthy of respect because, despite not being able to claim rights, it possesses intristic value. Therefore, the man has a duty towards the animal to consider everything that allows the promote their welfare during experimentation, not only to ensure the relability of the experimental results.

Animal testing is justified by the impossibility of being able to test everything on humans, in order to be able to carry out teratogenesis studies and also for the practical advantage of having results in a shorter time thanks to the shorter average life of common laboratory animals. The results are described in the scientific literature and it is important to remember that in the last century 32 Nobel Prizes were obtained thanks to animal testing [23]. However, this matter cannot be without rigorous regulation. Already in 1959, researchers Russell and Burch coined the rule of the “3Rs”: refinement, reduction and replacement to indicate, respectively, the need to select the least painful experimental procedures, reduce the number of animals to the indispensable and, if possible, to resort to experimental models or non-animal biological materials.

The functions of the ethics committee are mainly three: review and approval of the project, inspection of the treatment of animals, and veterinary support.

We followed the principles of animal care stated in the specific Italian law (D.L. 116/92). The study was approved by the local ethics committee and registered with the Department of Public Veterinary Health of the Ministry of Health.

## 6. Conclusions

Experimental urethroplasty performed in animal models provides a valuable opportunity to refine microsurgical skills and to validate new reconstructive techniques before their clinical application. The use of small laboratory animals such as rats and rabbits allows researchers to reproduce, on a manageable scale, the anatomical and technical challenges encountered in human urethral surgery. Microsurgical training in this context enhances precision, manual dexterity, and familiarity with delicate tissue handling. Equally important, it fosters an awareness of the ethical dimension inherent in all experimental research involving living beings. Successful outcomes in experimental surgery depend not only on technical ability but also on a rigorous scientific and ethical approach, from study design to postoperative care. Respect for animal welfare, meticulous surgical planning, and accurate reporting of methods and results are essential for the reliability and reproducibility of research findings. Ultimately, laboratory models contribute to advancing reconstructive urology by enabling safe and controlled development of new techniques and materials. The principles learned through these experiments—accuracy, respect, and responsibility—are the same that underpin excellence in clinical practice.

## Data Availability

Data can be found at the Ethics Committee, Center for Advanced Biotechnology, Cancer Institute, Largo Rosanna Benzi 10, 16132, Genova, Italy.
